# Perioperative Circulating Tumor Cells (CTCs), MCTCs, and CTC-White Blood Cells Detected by a Size-Based Platform Predict Prognosis in Renal Cell Carcinoma

**DOI:** 10.1155/2021/9956142

**Published:** 2021-10-25

**Authors:** Zhenlong Wang, Peng Zhang, Yue Chong, Yuquan Xue, Xiaojie Yang, Hecheng Li, Li Wang, Yaping Zhang, Qi Chen, Zhaolun Li, Li Xue, HongLiang Li, Tie Chong

**Affiliations:** ^1^Department of Urology, The Second Affiliated Hospital of Xi'an Jiaotong University, No. 157 Xiwu Road, Xi'an, Shanxi 710004, China; ^2^Department of Urology, The First Affiliated Hospital of Xi'an Jiaotong University, No. 277 Yanta West Road, Xi'an, Shanxi 710061, China

## Abstract

To explore the clinical significance of the perioperative counts of circulating tumor cells (CTCs), mesenchymal CTCs (MCTCs), and CTC- white blood cells (WBCs) in renal cell carcinoma patients. A total of 131 patients with renal cancer who underwent operation excision from our hospital were enrolled. In addition, 20 patients with benign renal diseases were recruited as a control. Blood samples were collected from the 131 patients, before operation and 3 months after surgery. Samples were also obtained simultaneously from the control group. CanPatrol CTC detection technique was used to enrich and identify CTCs, MCTCs, and CTC-WBCs. All enrolled patients were T1-3N0M0. From these, 52 patients with renal cancer underwent radical resection, while other 79 patients underwent nephron-sparing surgery. The positive rate of CTC, MCTC, and CTC-WBC before surgery were 95.4% (125/131), 61.1% (80/131), and 11.5% (15/131), respectively. Preoperative total CTCs, MCTCs, or CTC-WBCs were poorly correlated with patients' parameters. Preoperative CTC, MCTC, or CTC-WBC showed no association with progression-free survival (PFS). In contrast, postoperative total CTCs (≥6), positive MCTCs, and positive CTC-WBCs significantly correlated with recurrence and metastasis. These results remained independent indicators for worse PFS. In addition, the increased CTC and MCTC count after surgery also correlated with unfavorable PFS. The detection of six or more total CTCs, MCTC, or CTC-WBCs in peripheral blood after surgery might help to identify a subset of patients that have higher recurrent risk than the overall population of patients with at different stages of renal cancer.

## 1. Introduction

Worldwide, renal cancer represents one of the 10 most frequently diagnosed cancers in adults, accounting for 5% in men and 3% in women of all cancer diagnoses [1]. Renal cancer is an extremely invasive disease and benefits poorly from adjuvant chemotherapy or radiation therapy [2]. Surgical resection remains an effective therapy for clinical localized renal cancer, with options including radical nephrectomy and nephron-sparing surgery [3]. Importantly, 20% to 30% of patients with localized renal cancer experience disease recurrence or develop metastases after surgical excision. The median time to relapse after surgery is 1 to 2 years, with most relapse occurring within 3 years [4]. Even in patients considered to be potentially curable by surgery, metastasis can occur in 5-10 years. It is urgent to find biomarkers for better risk stratification of patients with renal cancer, which might allow identification of patients with an elevated risk of recurrence after nephrectomy. Our previous study showed that autophagy–related protein 7 (ATG7) be a new biomarker for the targeted therapy of renal cancer [5]. A recent report revealed that circulating tumor cells (CTCs) circulate in the blood and are believed to be vital seeds for hematogeneous tumor metastasis [6]. Evidence has shown that CTC counts have clinical relevance as a surrogate biomarker to noninvasive monitor for cancer progression and therapeutic decision-making [7–12]. In patients with early-stage hepatocellular carcinoma, higher CTC levels were correlated with early relapse. In addition, the epithelial cells can disseminate from tumors and penetrate into blood vessels by epithelial-mesenchymal transition (EMT) [13]. Hence, CTCs may be classified into three types: epithelial, mesenchymal (MCTC), and epithelial/mesenchymal hybrids. Increased MCTC count has been reported as a predictor of disease progression in breast cancer [14].

While single CTC has long been postulated to be travel solitarily in the bloodstream, recent studies reported the crosstalk between CTCs and the blood microenvironment. CTCs were found to form clusters and closely interacted with endothelial cells or platelets. It also works with macrophages and neutrophils to resist the physical stress in the circulation to evade the immune system and enable metastasis [15, 16]. Szczerba et al. [17] found that in patients with advanced breast cancer and in breast cancer mouse models, CTCs were associated white blood cells (WBCs). Patients with breast cancer harboring CTC- WBC clusters showed worse progression-free survival (PFS) compared with patients with no CTC-WBC clusters or less than five CTCs. The presence of CTC-WBC clusters was found to be a factor corresponding to poor prognosis in advanced breast cancer.

So far, publication reporting CTC data for renal cancer have relied mostly on small patient cohorts of different disease stages using various CTC-determination techniques, and they have shown few results regarding the correlation between CTC phenotypes, CTC-WBC clusters, and PFS in early stage of renal cancer [18–22]. In the present study, we evaluated CTCs in the peripheral blood obtained from patients with renal cancer by using the filtration method for CTC capture. A tri-color mRNA in situ hybridization (ISH) assay was conducted to identify and classify CTCs [23]. Our goal was to investigate the clinical significance of CTCs and CTC-WBC regarding PFS in operable patients with renal cancer. Better risk stratification of patients with renal cancer could help in identifying a subset of patients that might have higher recurrent risk than the overall population of patients.

## 2. Patients and Methods

### 2.1. Patient Samples

This study was enrolled 131 consecutive patients with renal cancer (tumor, node, and metastasis, TNM, and T_1-3_N_0_M0 stage) who underwent surgery from January 2015 to January 2020 at the Second Affiliated Hospital of Xi'an Jiaotong University, China. These renal cancer patients were confirmed by histological pathologist after surgery. This study protocol was reviewed and approved by the review board and ethics committee of the Second Affiliated Hospital of Xi'an Jiaotong University (approval #:2018021). Informed consent was obtained from all participants. Peripheral blood samples were obtained from all patients before and 3 months after surgery. In addition, blood specimens were collected from 20 patients with benign renal diseases as control group. The first patient follow-up of patients was performed at 3 months after surgery, then every 3 ~ 6 months for the first two years and every 6–12 months thereafter. The follow-up intervals were assessed more frequently if recurrence was suspected. The follow-up time ranged from 6 to 61 months. PFS was defined as the time from surgery to diagnosis of local recurrence, distant metastasis, or last follow-up.

### 2.2. Isolation of CTCs Using the CanPatrol System and Tricolor RNA-ISH Assay

The strategy used for both enrichment and characterization of renal cancer CTCs has been described in the previously published report [22, 23]. Peripheral blood samples (5 ml) were collected before surgery and 3 months after surgery. Processing was performed within 4 hours of collection. The samples were centrifuged for 5 minutes at 1500 rpm and to remove plasma. CTCs were further separated by using CanPatrol CTC enrichment technique (SurExam, Guangzhou, China). Briefly, red blood cells were removed by red cell lysis buffer from whole blood of the patients. CTCs were enriched via a filtration system.

**Figure 1:**
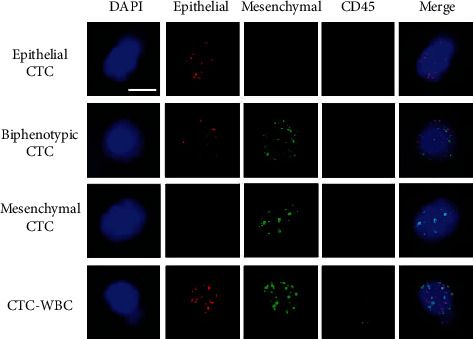
EMT phenotypes of CTCs and CTC-WBC were detected by the RNA in situ hybridization in renal cancer patients.

A tri-color RNA ISH assay was used to characterize CTCs and CTC-WBC clusters, including epithelial markers (EpCAM, CK8/18/19, labeled with Alexa Fluor 594), mesenchymal markers (Vimentin and Twist, labeled with Alexa Fluor 488), WBC marker (labeled by Alexa Fluor 750 conjugated anti-CD45), and nuclear marker (DAPI). CD45 was only expressed in leukocytes and not in tumor cells. CTCs were identified as epithelial and/or mesenchymal marker-positive DAPI+CD45-. CTC-WBC clusters were identified as one CTC with one/two-associated WBCs (representative pictures are shown in Figure 1).

### 2.3. Statistical Analysis

The association of preoperative CTC level and CTC-WBC positivity and clinicopathological parameters was performed by the chi-square test. PFS was defined as the time from the date of surgery to the date of progression (local recurrence or distant metastasis) or censored at the date of last follow-up. PFS was calculated by the Kaplan–Meier method and compared with the log-rank test. Univariate and multivariate analyses were performed using Cox's proportional hazards regression model with a forward stepwise procedure. Analyses were performed using the statistical software package IBM SPSS Statistics version 21.0 (IBM Corp., Armonk, NY, USA). All two-sided *p* values less than 0.05 were considered to be significant.

## 3. Results

### 3.1. Patient Characteristics

The study included a total of 131 renal cancer patients with renal cancer (T_1-3_N_0_M_0_). The clinicopathological features of the study cohort are summarized in Table 1, including age, sex, histology, differentiation, differentiation grade, TNM, stage, surgery types, and renal score. Most patients were diagnosed as having renal clear-cell carcinoma (113 cases, 86.3%), while the remaining had other types of renal cancer (18 cases, 13.7%). TNM stage classification showed that 112 (85.5%) patients had T1, 15 (11.5%) had T2, and 4 (3.0%) had T3 stage.

### 3.2. Identification of CTC Subtypes and CTC-WBC in Renal Cancer Patients

Blood samples were obtained from 131 patients and enriched for CTCs using CanPatrol technique. Based on the EMT markers, the detected CTCs could be classified into three phenotypes: epithelial, biphenotypic, and MCTCs (Figure 1). In the present study, most CTCs were found unaccompanied by other cells. However, we also detected CTC-WBC clusters in 15 patients (11.4%) before surgery and 17 patients (13.0%) after surgery. This slight elevation of CTC-WBCs number after surgery may concern positive margin rate at different T stages. All patients at this study had clear resection and 0% positive margin rate. Three different types of CTCs and CTC-WBC clusters were depicted in Figure 1. No CTCs were detected in patients with benign renal disease. Baseline CTCs were detected (≥1/5 mL) in 125 out of 131 patients with renal cancer (95.4%) with a median of 6 CTCs/5 mL blood (range: 0-53/5 mL blood). The positive rate of mesenchymal CTCs (MCTC) before surgery in all patients was 61.1% (80/131) with a median of 1 MCTC/5 mL (range: 0-9/5 mL blood). CTC-WBC clusters were present in 11.5% (15/131) patients, ranging from 0-2/5 mL blood.

### 3.3. Relationship between CTCs and Patients' Characteristics

To better investigate the potential application of CTCs in clinical practice, patients were divided into two groups, according to the median number of total CTC counts (CTC < 6 or CTC ≥ 6), MCTCs (negative/positive), and CTC-WBCs (negative/positive), respectively. Of the 131 patients included in the CTC analysis, 58 (44%) had CTCs < 6, and 73 (56%) had CTC ≥ 6 at baseline. The preoperative blood CTC level showed no significant association with clinicopathological features (Table 1). Likewise, the preoperative and postoperative MCTCs showed that there were no significant differences in patient age, sex, pathological type, differentiation grade, and TNM stages. The positive rate of CTC-WBC clusters was 11.5% (15/131) and 13.0% (17/131) before and after surgery, respectively. Similarly, no significant differences between the existence of CTC-WBCs and clinicopathological characteristics were found.

### 3.4. Prognostic Significance of Perioperative CTC Counts and Subtypes

**Figure 2:**
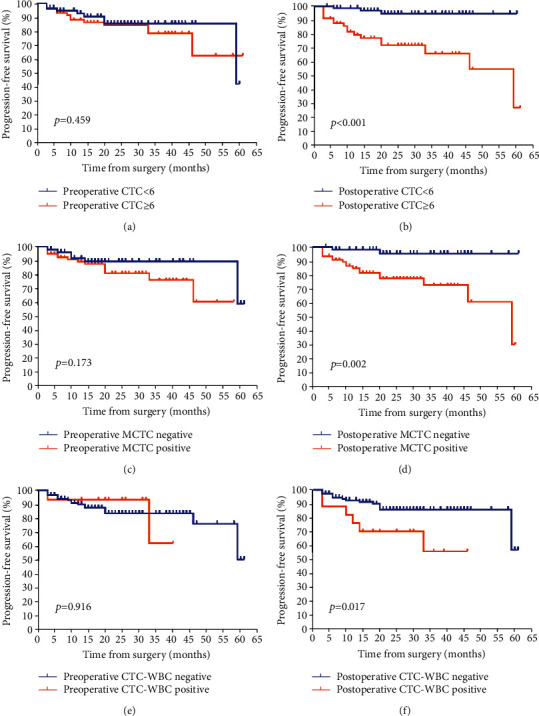
Kaplan-Meier curves for progression-free survival (PFS) of patients according to CTC, mesenchymal CTC (MCTC), and CTC-WBC before and after surgery. (a) Preoperative CTC and postoperative CTC (b) with PFS. (c) and (d) Preoperative MCTC and postoperative MCTC with PFS. (e) and (f) Preoperative CTC-WBC and postoperative CTC-WBC with PFS. *N* = 131. CTC: circulating tumor cells; WBC: white blood cell; PFS: progression-free survival; MCTC: mesenchymal CTC.

**Figure 3:**
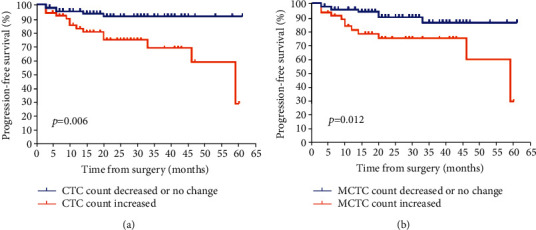
Kaplan-Meier curves for progression-free survival (PFS) of patients according to CTC or mesenchymal CTC (MCTC) variations before and after surgery. (a) Perioperative CTC variation and PFS and (b) perioperative MCTC variation and PFS. CTC: circulating tumor cells; PFS: progression-free survival; MCTC: mesenchymal CTC.

The follow-up duration of all patients was 6 to 61 months, with a median of 24 months. In total, 20 (15.3%) patients experienced a clinical relapse or developed metastasis, and 4 patients died of cancer by the end of follow-up. The Kaplan–Meier's survival curves revealed that patients with CTCs ≥ 6 after surgery had significantly poorer PFS (*p* < 0.001) than those with CTC < 6. Moreover, CTC count at baseline was found to have no significant correlation with PFS (*p* = 0.459, Figures 2(a) and 2(b)). Similarly, patients with the presence of MCTCs (per 5 mL blood) after surgery were more likely to have unfavorable PFS than those without MCTCs (*p* = 0.002, Figures 2(c) and 2(d)). In addition, the survival curves revealed that patients with/without CTC-WBC clusters before surgery exhibited no remarkable differences in PFS, while the negative of CTC-WBC after surgery was significantly associated with longer PFS (*p* = 0.916 and 0.017, respectively, Figures 2(e) and 2(f)). CTC counts after surgery were evaluated from all patients. Patients with increased CTC postoperation (compared with CTCs preoperation) categorized to have “increased CTCs,” while the other patients were categorized to have either “decreased CTCs” or “unchanged CTCs.” Similarly, these patients were also categorized according to the variations of MCTC during operation. The increase of CTCs or MCTCs was significantly associated with poorer PFS for patients with renal cancer, *p* = 0.006 and 0.012, respectively (Figures 3(a) and 3(b)).

### 3.5. Univariate and Multivariate Cox Regression Analysis

Univariate analyses showed that clinical factors significant for survival were T stage and surgical methods. CTC-related univariate analyses revealed significant association between postoperative CTCs/MCTCs/CTC-WBCs, CTCs/MCTCs changes, and PFS (all, *p* < 0.05) (Table 2). In a multivariate Cox regression analysis, after adjusting for the clinically significant univariate factor, postoperation (hazards ration [HR] 7.521, 95% confidence interval [CI] 2.065-27.397, *p* = 0.001), MCTC (HR 8.232, 95% CI 1.826-37.820, *p* = 0.006), and CTC-WBC (HR4.108, 95% CI 1.507-11.199, *p* = 0.006). CTC counts remained highly significant predictors of PFS. Patients with increased CTC (HR 2.784, 95% CI 1.001-7.743, *p* = 0.05) or MCTC (HR 2.329, 95% CI 0.901-6.021, *p* = 0.081) had slightly higher risk of progression compared with those CTC or MCTC decreased/unchanged group. We also further compared PFS of T1a vs. T1b-T3 stages via CTCs, MCTCs, and CTC-WBCs after surgery, respectively. There were no significant differences to be found (Table 2).

.

## 4. Discussion

CTCs are considered the pivotal component of the metastatic cascades and have only been extensively studied only in approximately the last decade [13, 24]. The clinical application of CTCs was assessed previously in well-validated studies. Their prognostic value was evaluated using cell search system, which depends on the expression of epithelial marker EpCAM at pretreatment baseline in advanced disease [7–9]. As for renal cancer, several published studies have also focused on CTCs in advanced stages with various techniques [22, 25]. Only a few studies assessed CTCs in patients with early-stage renal cancer [23]. In the present study, we detected total CTCs, EMT CTCs, and CTC-WBCs before and after surgery in T_1-3_N0M0 patients with renal cancer and found that these CTC subtypes had a significant clinical association with renal cancer progress predication.

As mentioned previously, CTCs in the bloodstream can be classified into three types, and their detection relied on a combination of membrane filtration and epithelial/mesenchymal biomarker-based identification. Recent reports indicated that the expression of EMT markers in CTCs is relevant process for invasion and metastasis in several cancers, such as breast, colorectal, nonsmall cell lung, gastric, and prostate cancers [26–30]. The CTC detection method in the present study allowed the isolation of pivotal EMT CTCs in renal cancer. In addition, CTCs in the bloodstream were found to migrate with leukocytes. The presence of CTC-WBCs correlates with shorter PFS in patients with breast cancer compared with the absence of CTC-WBCs or without CTC-WBC or total CTC ≥ 6. Besides, the transcriptome profiles of CTC-WBCs are different from those of other CTCs [17]. It is necessary to elucidate the role of CTC-WBC clusters in disease progression in more cancer types. Total CTCs, MCTCs, and CTC-WBC cluster detection could contribute to improving the accuracy and clinical implications of CTCs. We initially found that perioperative total CTCs, MCTCs, or CTC-WBCs showed minor differences regarding age, sex, T stages, or differentiation. Similar result was also found in nonmetastasis breast cancer, where preoperative CTCs were poorly associated with tumor size, grade, or lymph node status [31]. However, CTCs appear to provide important reference information regarding an individual patient's risk for relapse or progression. Bluemke et al. [18] discovered that CK-positive CTCs were significantly correlated with inferior overall survival (OS) of patients with renal cancer. Nel et al. [32] found that the presence and quantity of CD133-positive or N-cadherin-positive CTC were associated with poor PFS in 14 patients with renal cancer. In the present study, patients with higher MCTCs, both before and after surgery, were more likely to have a bad clinical outcome during follow-up. More than six CTCs and higher positive MCTCs postoperation were independent prognostic indicators for poorer PFS. The correlation between preoperative CTCs and diseases recurrence has shown to be controversial in different cancers. Baseline CTCs were significantly related with PFS and OS in colon cancer patients undergoing surgery, while in breast cancers, baseline CTCs were poorly related with recurrence or progression [31]. As for total CTCs and MCTCs postsurgery, similar results were found in several cancers [33, 34]. The clinical use of CTC clusters to monitor treatment response has been reported in other types of cancers [14, 35]. Patients who had a decreased total CTCs or MCTC count after surgery showed increased recurrence-free and a relatively longer disease-free survival period. Moreover, our study is in line with a previous study showing that the patients with lung cancers with increased CTC count after radiotherapy had a worse disease progression than those with low CTC count [36, 37]. Similarly, in hepatocellular cancers, patients with increased postoperative MCTC count relapse earlier than patients with low MCTC counts [38]. Thus, perioperative monitoring of CTC and MCTCs changes may provide another predictor of recurrence in addition to conventional clinical parameters.

Blood metastasis of cancer cells is very easy as only few CTCs are released daily in the bloodstream, ultimately surviving and establishing secondary lesions. In peripheral blood, CTCs can also migrate as cell clusters, named “circulating tumor microemboli” (CTMs), which may comprise leukocytes, endothelial cells, platelets, neutrophils, and other cells held together by cell adhesion proteins [39]. The close interaction between CTCs and nonmalignant cells in bloodstream may aid CTCs to evade the immune system and enable metastasis [15–17]. In addition, we found that patients positive for CTC-WBCs 3 months after surgery were characterized to be shorter PFS that those negative for CTC-WBCs. Similar to our findings, Szczerba et al. [17] showed that PFS in patients with positive CTC-WBC clusters was significantly shorter than patients with no CTC-WBC or patients with no less than five CTCs. They also found that CTCs from CTC-WBC clusters showed high *ARG1*, *CXCL2*, *CXCL10*, *CCL2*, *CXCR2*, and *VEGFA* expression. We previously showed that ATG7 is a critical biomarker for renal cancer prognosis [5]. In the current study, we showed that total CTCs, MCTCs, and CTC-WBCs also play pivotal roles in cancer progression.

In multivariate Cox regression analysis, total CTC, and the presence of MCTC and CTC-WBC after surgery remained independent as the independent predictors of PFS. Patients with increased CTCs or MCTCs had slightly higher risk of progression compared with patients with decreased/unchanged CTCs or MCTCs. These results further support the potential role of CTC monitoring for improved cancer management.

## 5. Conclusion

In conclusion, the current study evaluated the relationship between perioperative CTC subtypes and PFS in operable patients with renal cancer. Our findings demonstrated that higher postoperation total CTC (CTC ≥ 6), positive MCTCs, and positive CTC-WBC significantly correlated with recurrence or metastasis and remained independent indicators for worse PFS. These data supported that CTCs are the key biomarkers of renal cancer relapse after surgery.

## Figures and Tables

**Table 1:** Relationship of perioperative total CTCs, mesenchymal CTC, CTC-WBC, and clinical characteristics. CTCs: circulating tumor cells; WBC: white blood cells; TNM: tumor, node, metastasis; *n*: case number; *p*: statistical value.

Clinical parameters	*n*	Total CTC				Mesenchymal CTC				CTC-WBC			
		Presurgery		Postsurgery		Presurgery		Postsurgery		Presurgery		Postsurgery	
		CTC < 6	CTC ≥ 6	CTC < 6	CTC ≥ 6	Negative	Positive	Negative	Positive	Negative	Positive	Negative	Positive
Age													
<50	32	13	19	17	15	9	23	13	19	28	4	29	3
≥50	99	45	54	55	44	42	57	41	58	88	11	85	14
*p*			0.633		0.81		0.149		0.937		0.76		0.762
Sex													
Male	83	35	48	44	39	37	46	30	53	70	13	69	14
Female	48	23	25	28	20	14	34	24	24	46	2	45	3
*p*			0.523		0.555		0.081		0.121		0.051		0.107
Pathological subtypes													
Clear-cell carcinoma	113	52	61	60	53	47	66	45	68	100	13	98	15
Others	18	6	12	12	6	4	14	9	9	16	2	16	2
*p*			0.314		0.283		0.11		0.41		1		1
Differentiation													
Low	22	7	15	13	9	7	15	11	11	19	3	21	1
Middle	102	48	54	57	45	41	61	42	60	92	10	88	14
High	7	3	4	2	5	3	4	1	6	5	2	5	2
*p*			0.446		0.38		0.79		0.29		0.248		0.189
TNM stage													
T1	112	52	60	63	49	43	69	45	67	99	13	97	15
T2	15	4	11	6	9	5	10	6	9	13	2	13	2
T3	4	2	2	3	1	3	1	3	1	4	0	4	0
*p*			0.327		0.39		0.37		0.45		0.811		1
Types of surgery													
Nephron-sparing surgery	79	35	44	51	28	27	52	34	45	68	11	68	11
Radical excision	52	23	29	21	31	24	28	20	32	48	4	46	6
*p*			0.993		0.007		0.16		0.60		0.273		0.691
Renal score													
Low risk	28	12	16	17	11	7	21	13	15	24	4	24	4
Middle risk	72	32	40	42	30	31	41	32	40	63	9	66	6
High risk	31	14	17	13	18	13	18	9	22	29	2	24	7
*p*			0.983		0.243		0.23		0.28		0.576		0.126

Fluorescence microscopy images show three types of CTCs with positive expression of epithelial markers (EpCAM and CK8/18/19, red dots), biphenotypic markers (red dots and green dots), and mesenchymal markers (Vimentin and Twist, green dots). CTC-WBC was characterized as DAPI + CD45 + epithelial + and/or mesenchymal+. Scale bar = 10 *μ*m. EMT: epithelial-mesenchymal transition; CTC: circulating tumor cells; WBC: white blood cell; ISH: in situ hybridization.

**Table 2:** Univariate and multivariate Cox regression analysis for prediction of PFS.

Variable		Univariate Cox			Multivariate Cox∗	
	HR	95% CI	*p*	HR	95% CI	*p*
Preoperative CTC (CTC ≥ 6/CTC < 6)	1.398	0.569-3.438	0.465	——		
Postoperative CTC (CTC ≥ 6/CTC < 6)	7.803	2.278-26.728	0.001	7.521	2.065-27.397	0.001
CTC variation (elevated/not elevated)	3.481	1.329-9.119	0.011	2.784	1.001-7.743	0.05
Preoperative MCTC (positive/negative)	1.996	0.718-5.549	0.185	——		
Postoperative MCTC (positive/negative)	7.19	1.665-31.05	0.008	8.232	1.826-37.820	0.006
MCTC variation (elevated/not elevated)	3.006	1.218-7.42	0.017	2.329	0.901-6.021	0.081
Preoperative CTC-WBC (positive/negative)	0.925	0.212-4.029	0.917	——		
Postoperative CTC-WBC (positive/negative)	3.079	1.169-8.111	0.023	4.108	1.507-11.199	0.006
Postoperative CTCs (T1a vs. T1b-T3)	1.035	0.671-2.437	0.431			
Postoperative MCTCs (T1a vs. T1b-T3)	1.234	0.837-1.587	0.202			
Postoperative CTC-WBCs (T1a vs. T1b-T3)	1.463	0.734-2.978	0.200			

^∗^: adjusted by age, sex, T stage, types of surgery, and tumor differentiation. CTC: circulating tumor cells; MCTC: mesenchymal circulating tumor cell; PFS: progression-free survival; *p*: statistical value.

## Data Availability

The data used to support the findings of this study are available from the corresponding author upon request.
